# Emergence and Control of Zoonotic Viral Encephalitis

**DOI:** 10.3201/eid0908.030319

**Published:** 2003-08

**Authors:** Charles H. Calisher, Betty Dodet, Diane Griffin

**Affiliations:** * Colorado State University, Fort Collins, Colorado, USA; † Fondation Mérieux, Lyon Cedex, France; ‡ Johns Hopkins Bloomberg School of Public Health, Baltimore, Maryland, USA

More than 50 researchers and administrators from over a dozen countries attended a symposium on the emergence and control of zoonotic viral encephilitis. Held April 6–8, 2003, in a convivial setting at Les Pensières, Veyrier du Lac, near Annecy in the French Alps, this meeting was one of a series on the emergence and control of infectious diseases, sponsored and organized by the Mérieux Foundation. The general objectives were to review the biology of viral encephalitis, the virulence and genetic evolution of encephalitis viruses, and the factors involved in emergence of these diseases.

Emergence or reemergence of viruses may be due to virus evolution, to the impact and influence of human populations on previously undisturbed ecosystems, or to better recognition. Clearly, we must understand the basic mechanisms by which these viruses emerge or reemerge and cause illnesses. Methods for detecting infections caused by neurotropic viruses and for detecting viruses or their genome sequences are available and improving. Methods for detecting antibodies also have improved.

Examples of recently recognized viruses causing encephalitis in humans, livestock, or wildlife include Hendra and Nipah viruses (henipaviruses; family *Paramyxoviridae*, genus *Henipavirus*), both of which are neurotropic, and Australian bat lyssavirus (family *Rhabdoviridae*, genus *Lyssavirus*), also a neurotrope. All three viruses, and others related to them, have been shown to have fruit bats (*Pteropus* spp.) as their natural hosts. Progress is being made in understanding transcription regulation and cell fusion by henipaviruses. In addition, basic epidemiologic procedures and classical prevention strategies have been put into place to prevent infections caused by these viruses.

Since 1988, a worldwide effort has been under way to eradicate the nonzoonotic but encephalitogenic poliomyelitis viruses. The number of cases has been reduced by 99%, and the natural occurrence of these viruses now is limited to seven countries. The system established to conduct surveillance and response may provide a model for use in tracking and controlling other viruses causing encephalitis. Long-term studies of ecologic parameters, seasonality, and changing virus and vector prevalence rates are being used to determine risk factors in various arbovirus infections, including Japanese encephalitis virus in Thailand, and are being applied for prevention and control.

In Russia, where West Nile virus has long been recognized but has not caused any major diseases, recent detection of various virus genotypes suggests a mélange of genotypes circulating in various areas and transported between areas by birds. Generation and maintenance of continuous genetic variation may lead to partial protection and escape mutants, which could provide a “pump” that generates more variants and “new” viruses. When these genotypes adapt to naïve populations of birds, horses, and humans, in the presence of competent arthropod vectors, epidemics may arise and new opportunities for these viruses and virus variants may occur, perhaps including West Nile virus into the New World in 1999. Evidence presented suggests that little genomic variation in New World West Nile virus has occurred since its 1999 recognition there; this situation is likely to change.

Continuing to make the classical epidemiologic observations that have characterized disease investigations in the previous half-century is important. However, to understand the overall effects of virus outbreaks, denominators are needed. Numerous presentations demonstrated that we are beginning to understand the molecular mechanisms leading to pathogenetic events. Further studies may provide information useful for the development of antiviral compounds and candidate vaccines.

Attendees were provided with an overview of various transmission cycles of arboviruses, which are concomitantly diverse in regards to their hosts and vectors. Viral neuroinvasiveness appears to depend on the uniqueness of phylogenetically diverse hosts, their ages, genetic predispositions, immune status, virus origin, passage level, dose, and other factors—a complex situation to investigate and comprehend. Critical factors impacting neuroinvasiveness and neuronotropism must be coupled to cause encephalitis. Viral mutations may affect the ability of the virus to replicate in cells, altering viral virulence; however, specific genomic and polyprotein sequence changes may account for the high viremias and replication in neurons that are central to emergence. The extent of the roles of various proteins in virus infections, neuronal involvement, and apoptosis are being recognized. Now we are beginning to understand complex signaling mechanisms, antibody-producing cell types, cytokines, and the cellular responses and pathways leading to both disease and protection from disease.

Considerable progress has been made in understanding the relationships between genetic and functional diversities, neuronal receptors, transport, and cellular protein-virus interactions. Such understanding is critical to further insights to neurotropism, pathogenesis, pathogenetic mechanisms, and immunogenicity.

Phylogenetic trees were used to describe the evolution of encephalitic flaviviruses, geographic exclusion, virus persistence, and flaviviral recombination as a mechanism of flaviviral evolution. In addition, data were presented that illustrated the persistence of, and immune modulation by, alphaviruses, which, in concert, allow the virus to replicate while preventing the host from responding to its benefit.

Other than the classical techniques of preventing infection, little was mentioned about disease control during this symposium. Control must be based on rapid recognition of early cases, subsequent immunization of persons or animals at risk, or immunization of persons or animals with the potential to be at risk, such as travelers, laboratory personnel, and attending clinicians. Attendees learned about diverse methods being used to develop vaccines. Representatives from the World Health Organization explained that organization’s plans for responding to disease emergence and for preventing zoonotic diseases from reaching human populations.

New paradigms for field studies of zoonotic diseases are necessary. These approaches must include longitudinal and in-depth investigations of agent, host, habitat, and environment if we are to predict risk and respond in an appropriate manner. At this time, zoonotic disease control comprises prevention and public education and not much more. Progress is being made in rapid diagnosis, production of sophisticated vaccines, and understanding of the molecular mechanisms by which zoonotic viruses persist and cause disease.

